# The immunometabolic tipping point: forecasting the systemic shift from psoriasis to psoriatic arthritis

**DOI:** 10.3389/fimmu.2026.1944289

**Published:** 2026-09-23

**Authors:** Xi Chen, Can Chen, Jianqiang Yang

**Affiliations:** 1 First Affiliated Hospital of Huzhou University, Huzhou, China; 2 Huzhou Central Hospital, Fifth School of Clinical Medicine of Zhejiang Chinese Medical University, Huzhou, China; 3 Huzhou Central Hospital, Affiliated Central Hospital of Huzhou University, Huzhou, China

**Keywords:** immunometabolism, multi-omics, predictive biomarkers, psoriasis, psoriatic arthritis

## Abstract

Psoriasis (PsO) is a chronic immune-mediated inflammatory disease that progresses to psoriatic arthritis (PsA) in up to 30% of patients. The transition from localized cutaneous inflammation to systemic articular involvement represents a critical window for early intervention; however, accurately predicting which patients will develop PsA remains a significant clinical challenge. Growing evidence highlights immunometabolic dysregulation as a central driver of this systemic shift. The progression from PsO to PsA is fueled by a complex interplay between immune dysregulation—centered on the IL-23/Th17 axis, tissue-resident memory T cell recirculation, and regulatory T cell dysfunction—and systemic metabolic disturbances, including obesity, insulin resistance, dyslipidemia, and adipokine imbalance. This review comprehensively examines the immunometabolic mechanisms bridging skin and joint pathology and evaluates emerging candidate biomarkers for predicting disease progression. Promising indicators include immunological markers, such as declining serum CXCL10 levels and circulating skin-homing CD8+CCR10+ T cells, as well as metabolic signatures, including an elevated leptin-to-adiponectin ratio and distinct lipidomic and metabolomic profiles. Given the multifactorial nature of psoriatic disease, individual biomarkers often lack sufficient predictive power. Therefore, we highlight the paradigm shift toward multi-omics integration. Leveraging machine learning to synthesize genomic, transcriptomic, proteomic, metabolomic, and epigenomic data offers a powerful strategy for developing robust predictive models. The identification and validation of integrated immunometabolic biomarker profiles will facilitate early risk stratification, enable preemptive disease-modifying and lifestyle interventions, and advance precision medicine in the management of psoriatic disease.

## 1 Introduction

Psoriasis (PsO) is a chronic immune-mediated inflammatory skin disease affecting approximately 2-3% of the global population, characterized by hyperproliferation of keratinocytes and infiltration of immune cells into the dermis and epidermis (1, 2). In up to 30% of patients, PsO progresses to psoriatic arthritis (PsA), a heterogeneous inflammatory arthropathy involving peripheral joints, entheses, and the axial skeleton (3, 4). The transition from cutaneous PsO to articular involvement represents a critical juncture in disease progression, often resulting in irreversible joint damage, functional impairment, and diminished quality of life if not recognized and treated early (5, 6).

The concept of psoriatic disease has emerged to capture the systemic nature of this condition, encompassing not only skin and joint manifestations but also a spectrum of associated comorbidities including metabolic syndrome, cardiovascular disease, obesity, and type 2 diabetes mellitus (2, 7, 8). This systemic dimension underscores the inadequacy of viewing PsO as a disorder confined to the skin. Rather, PsO represents a chronic inflammatory state with profound metabolic consequences, and conversely, metabolic dysfunction may actively drive inflammatory progression (9, 10).

The IL-23/IL-17 axis constitutes the central immunopathogenic pathway in psoriatic disease, with TNF-α serving as a critical amplifier of inflammation (1, 11, 12). Genetic studies have identified risk loci such as HLA-Cw6, IL23R, and TRAF3IP2 that predispose individuals to PsO, while distinct genetic signatures including HLA-B27 appear to confer additional risk for PsA development (13–15). However, genetic susceptibility alone cannot fully explain disease progression, and growing evidence points toward immunometabolic mechanisms as key determinants of the transition from PsO to PsA (16, 17).

Immunometabolism has emerged as a crucial framework for understanding chronic inflammatory diseases, but it must be viewed as a bidirectional and multi-scalar phenomenon (9, 18). At the cellular level, it involves intrinsic metabolic reprogramming—such as increased glycolysis, glutaminolysis, and fatty acid synthesis—required to fuel the effector functions of activated T cells, particularly Th17 cells (9, 17). At the systemic level, immunometabolism encompasses the continuous cross-talk between immune responses and metabolic organs. This relationship is inherently two-way: systemic metabolic shifts can dictate immune polarization (metabolism-to-immune), while chronic immune activation can conversely induce systemic metabolic dysfunction (immune-to-metabolism). In psoriatic disease, the collision of these cellular demands and systemic alterations creates a vicious feed-forward loop that perpetuates inflammation and actively facilitates the extension from skin to joint pathology (19–21).

Identifying biomarkers capable of predicting which PsO patients will develop PsA remains a critical unmet need (5, 22). Current clinical prediction tools lack sufficient accuracy, and diagnostic delays averaging several years remain common (4, 5). The integration of immunological and metabolic biomarkers offers a promising avenue for improving risk stratification and enabling early intervention strategies (23, 24). In the context of this review, it is crucial to explicitly define our framework for biomarkers. We define a “predictive biomarker” not merely as a diagnostic indicator of established disease, but as a quantifiable immunological or metabolic parameter capable of forecasting the future onset of articular involvement in patients currently presenting with skin-only PsO. Unlike cross-sectional disease-activity markers that reflect current inflammatory burdens, true predictive biomarkers capture the dynamic “immunometabolic tipping point”—the precise molecular transition from local cutaneous inflammation to systemic disease. Therefore, the overarching aim of this review is inherently translational, bridging basic pathophysiology with clinical application. First, we dissect the bidirectional immunometabolic interactions that drive the systemic shift from localized PsO to articular PsA. Second, we evaluate how a deep understanding of these foundational mechanisms directly informs the identification, multi-omics integration, and future validation of predictive biomarker profiles. By framing biomarker discovery through the lens of immunometabolism, we aim to provide a comprehensive roadmap for advancing precision medicine and early disease interception in psoriatic disease.

## 2 Immunometabolic dysregulation as a driver of systemic disease in PsO

The progression from localized cutaneous inflammation to systemic disease involving joints, entheses, and metabolic tissues reflects a fundamental shift in the nature and distribution of immune dysregulation. In PsO, the inflammatory milieu is initially dominated by resident memory T cells and keratinocyte-derived cytokines within skin lesions (3, 9). However, as disease persists, systemic immune activation becomes evident, characterized by elevated circulating pro-inflammatory cytokines, altered adipokine profiles, and metabolic disturbances that extend beyond the skin (2, 7, 10) (**Figure 1**).

**Figure 1:**
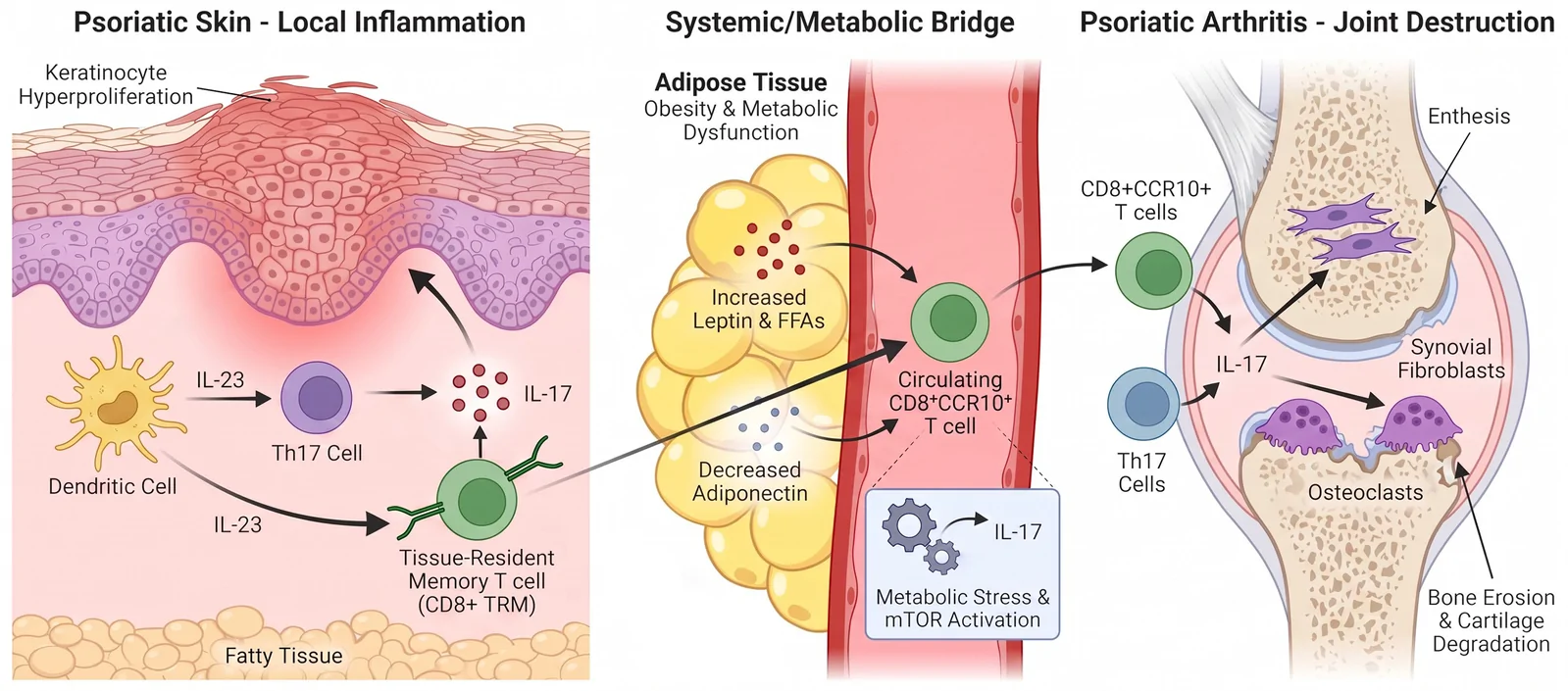
Immunometabolic mechanisms driving the transition from localized cutaneous psoriasis to systemic psoriatic arthritis. (Left) In psoriatic skin, local inflammation is characterized by keratinocyte hyperproliferation. Dendritic cell-derived IL-23 stimulates T helper 17 (Th17) cells and CD8+ tissue-resident memory T (TRM) cells to secrete IL-17, perpetuating cutaneous inflammation. (Middle) The systemic and metabolic bridge is heavily influenced by obesity-driven adipose tissue dysfunction, resulting in increased levels of leptin and free fatty acids (FFAs) alongside decreased adiponectin. This metabolic stress, coupled with mechanistic target of rapamycin (mTOR) activation, exacerbates systemic immune dysregulation and promotes IL-17 production. Concurrently, skin-resident CD8+ TRM cells acquire the capacity to recirculate, entering the bloodstream as circulating skin-homing CD8+CCR10+ T cells. (Right) Upon homing to the joints and entheses, these recirculating CD8+CCR10+ T cells and Th17 cells produce abundant IL-17. This key cytokine targets synovial fibroblasts and osteoclasts, ultimately leading to the hallmark joint destruction, bone erosion, and cartilage degradation observed in psoriatic arthritis.

Tissue-resident memory T (TRM) cells play a central role in maintaining chronic inflammation in psoriatic skin. These cells persist in the epidermis and dermis after clinical resolution of lesions, providing a reservoir for disease recurrence (3, 25). CD8+ TRM cells expressing CD103 and CD49a are enriched in psoriatic skin and produce IL-17 and IFN-γ upon stimulation (3, 25). TRM cells can recirculate and potentially home to other tissue sites, including joints and entheses, providing a mechanistic link between cutaneous and articular disease (3, 25). Leijten et al. demonstrated that CD8+CCR10+ T cells, which share transcriptional features with skin-resident memory T cells, are expanded in the circulation of PsA patients compared to those with PsO alone, suggesting that aberrations in cutaneous tissue homeostasis contribute to arthritis development (25).

Regulatory T cells (Tregs) exhibit functional impairments in psoriatic disease. In psoriatic skin lesions, Tregs are present but display reduced suppressive capacity and may even acquire pro-inflammatory properties (3). This Treg dysfunction contributes to the breakdown of immune tolerance and the perpetuation of inflammation. The balance between Tregs and effector T cells, particularly Th17 and CD8+ TRM cells, is heavily skewed toward inflammation in progressive disease (3, 17). As the disease advances, the marked expansion of pathogenic CD8+ T cell subsets (both tissue-resident and circulating) drastically overwhelms the regulatory capacity of Tregs, leading to a notably decreased Treg/CD8+ ratio. Crucially, successful treatment with IL-23 inhibitors has been shown to restore this vital immunological homeostasis. Specifically, these targeted therapies facilitate a significant increase in the Treg to CD8+ TRM ratio, reversing the pathogenic imbalance. This robust restoration of the Treg/CD8+ equilibrium underscores that targeting this specific cellular dynamic is therapeutically essential for halting systemic progression (3).

The IL-23/Th17 axis represents the dominant pathogenic pathway in psoriatic disease. IL-23, produced by dendritic cells and macrophages, stabilizes and expands Th17 cells, which secrete IL-17A, IL-17F, and IL-22 (1, 11, 12). IL-17 acts on keratinocytes, synovial fibroblasts, and osteoclasts, driving tissue inflammation and remodeling (12, 26). The central role of this pathway is underscored by the clinical efficacy of IL-17 and IL-23 inhibitors in both PsO and PsA (11, 26). However, IL-23-independent pathways of IL-17 production also exist, involving cytokines such as IL-7 and IL-9, and may be particularly relevant in the context of metabolic stress (27, 28).

Metabolic stress profoundly influences T cell differentiation and function. In the presence of obesity and insulin resistance, elevated levels of leptin, free fatty acids, and glucose create a pro-inflammatory milieu that promotes Th17 polarization and IL-17 production (19, 28). Stober et al. demonstrated that metabolic stress expands polyfunctional, pro-inflammatory Th17 cells in PsA patients, and that IL-17 production can occur independently of IL-23 under these conditions (28). This finding has therapeutic implications, as it may explain why some patients respond better to IL-17 inhibitors than to IL-23 inhibitors (28).

The mechanistic link between metabolic dysfunction and immune activation is mediated in part through the mTOR signaling pathway. mTOR integrates signals from growth factors, nutrients, and energy status to regulate cell growth, proliferation, and metabolism (9, 20). In psoriatic disease, sustained mTOR activation promotes keratinocyte hyperproliferation, Th17 differentiation, and pro-inflammatory cytokine production (9, 20). mTOR activation has also been linked to the transition from PsO to PsA and to cardiovascular complications, suggesting that this pathway represents a nexus connecting inflammation and metabolism (20).

Conversely, the immune-to-metabolism pathway illustrates how localized psoriatic inflammation fundamentally alters systemic metabolic homeostasis. The chronic overproduction of pro-inflammatory cytokines—predominantly TNF-α, IL-17, and IL-6—from psoriatic skin and recirculating immune cells spills into the systemic circulation, profoundly affecting metabolic organs. For instance, circulating TNF-α and IL-17 directly disrupt insulin receptor substrate-1 (IRS-1) signaling in skeletal muscle, adipose tissue, and the liver, thereby inducing systemic insulin resistance. Furthermore, this cytokine storm impairs lipid handling within adipocytes and hepatocytes, leading to elevated circulating free fatty acids and characteristic dyslipidemia. Consequently, the cutaneous immune response acts as a central generator of systemic metabolic syndrome, which in turn feeds back to exacerbate the disease.

Adipose tissue functions as an active endocrine organ, secreting a wide array of adipokines that modulate immune responses (19, 21, 29). In obesity, adipose tissue becomes inflamed, with infiltration of macrophages and T cells, leading to altered adipokine secretion characterized by increased leptin, resistin, and visfatin, and decreased adiponectin (19, 21, 29). Leptin promotes Th17 responses and inhibits Treg function, while adiponectin exerts anti-inflammatory effects (21, 29). This adipokine imbalance contributes to the low-grade systemic inflammation that characterizes obesity and may facilitate the progression from PsO to PsA (19, 21).

The relationship between obesity and PsA is bidirectional and clinically significant. Obesity is not only a risk factor for PsA development but also predicts more severe disease, poorer treatment responses, and increased cardiovascular morbidity (6, 19, 30, 31). Weight loss interventions have been shown to improve disease activity in PsA, supporting a causal role for adiposity in driving inflammation (6, 32, 33). Very low-calorie ketogenic diets lead to rapid improvements in disease activity, inflammatory biomarkers, and cardiovascular risk parameters, further emphasizing the importance of metabolic interventions in PsA management (33).

## 3 Immunological biomarkers predictive of PsA onset

Identifying patients with PsO who are at high risk of developing PsA remains a major clinical challenge. Building upon the mechanistic framework discussed above, it becomes evident that predictive biomarkers are not merely random clinical correlates, but direct molecular readouts of the underlying immunometabolic tipping point. Immunological biomarkers that capture the transition from cutaneous to articular inflammation hold promise for improving risk stratification and enabling early intervention (23, 24). Several candidate biomarkers have emerged from studies comparing immune profiles in PsO patients who do and do not develop PsA.

T cell subsets, particularly CD8+ T cells, have garnered attention as potential predictive biomarkers. CD8+CCR10+ T cells, which co-express skin-homing receptors CCR4 and cutaneous lymphocyte antigen, are enriched in the circulation of PsA patients compared to those with PsO alone (25). These cells exhibit a Tc2/22-like cytokine profile, produce IL-17 and IL-22, and display transcriptional features of TRM cells (25). The presence of these cells in the circulation may reflect the recirculation of skin-derived TRM cells that have acquired the capacity to home to joints and entheses (3, 25). Longitudinal studies are needed to determine whether expansion of CD8+CCR10+ T cells precedes the clinical onset of PsA.

Cytokine profiling has revealed differences between PsO and PsA that may have predictive value. Ruscitti et al. compared serum levels of 20 biomolecules in treatment-naïve patients with PsO, PsA with PsO, and PsA sine PsO (34). While the overall cytokine profiles were broadly similar across groups, supporting the concept of a psoriatic disease continuum, some differences emerged. Patients with PsO showed elevated E-selectin and IL-8 levels, while those with PsA and PsO had higher ICAM-1 levels (34). Patients with extensive skin disease (Psoriasis Area and Severity Index [PASI] ≥ 10) exhibited enhanced levels of IL-8, TNF-α, E-selectin, MIP-1α, and MIP-1β, suggesting that the burden of cutaneous inflammation influences the systemic inflammatory milieu (34).

The chemokine CXCL10 has emerged as a candidate biomarker for PsA development. CXCL10 is elevated in psoriatic affected tissues and serum, and Abji et al. demonstrated that serum CXCL10 levels decline over time in PsO patients who subsequently develop PsA (35). In a longitudinal study of PsO patients without arthritis who were followed prospectively, CXCL10 levels showed a significant downward trend in converters prior to PsA onset, while non-converters maintained stable levels (35). This pattern suggests that CXCL10 may be consumed or sequestered at sites of developing inflammation, and declining levels could serve as an early warning signal for impending articular involvement (35). From an immunometabolic perspective, CXCL10 not only regulates immune cell homing to the joints but also critically mediates the recruitment of pro-inflammatory macrophages into adipose tissue (36). This CXCL10-driven adipose infiltration actively promotes localized adipocyte dysfunction and subsequent systemic insulin resistance, tightly linking chemokine activity to metabolic deterioration (37).

Type I interferon pathway activity distinguishes PsA from other inflammatory arthritides and may have predictive value. Fragoulis et al. found that despite increased DNA damage accumulation in peripheral blood mononuclear cells (PBMCs) from PsA patients, the type I interferon (IFN-I) score was lower in PsA compared to healthy controls (38). This IFN-I downregulation contrasts with findings in systemic lupus erythematosus (SLE) and rheumatoid arthritis (RA), where DNA damage is associated with IFN-I pathway activation (38). The unexpected IFN-I suppression in PsA may reflect the operation of other cytokines, such as IL-17 and TNF-α, that suppress IFN-I responses, and could be exploited as a diagnostic or predictive biomarker (38).

Inflammasome activation and associated proteins represent another class of candidate biomarkers. Forouzandeh et al. demonstrated that serum levels of apoptosis-associated speck-like protein containing a caspase-recruitment domain (ASC) and IL-18 were significantly elevated in PsO patients compared to healthy controls, with ASC showing discriminatory performance (AUC 0.922) (39). Crucially, these inflammasome-associated proteins actively perturb systemic metabolism. The inflammasome itself serves as a major intracellular sensor for metabolic “danger signals,” such as soluble urate, ceramides, and saturated fatty acids. Once activated, the resultant overproduction of IL-18 heavily impairs systemic metabolic homeostasis by interfering with insulin signaling cascades in peripheral tissues and promoting hepatic steatosis (39). Thus, elevated ASC and IL-18 are not mere immunological bystanders, but active drivers of the metabolism-to-immune feed-forward loop in progressing psoriatic disease.

Calprotectin (S100A8/A9) is an established inflammatory marker in RA, but its role in PsA is less clear. Røym et al. found that plasma calprotectin levels were elevated in PsA patients compared to controls and decreased with treatment, but showed no clinically significant correlation with ultrasound-detected synovitis (40). This finding suggests that calprotectin may reflect systemic inflammation rather than localized joint inflammation in PsA, limiting its utility as a specific biomarker for articular involvement (40).

Protein biomarkers classically associated with disease activity across PsA domains—such as serum amyloid A (SAA), IL-6, YKL-40, and ICAM-1—also exert profound effects on systemic metabolism (41). Beyond reflecting joint inflammation, these proteins are active participants in the “immune-to-metabolism” pathway (42). For example, IL-6 powerfully disrupts hepatic insulin sensitivity and stimulates unrestrained lipolysis (43). Simultaneously, SAA incorporates into high-density lipoprotein (HDL) particles, displacing apolipoprotein A-I and transforming HDL from a protective molecule into a dysfunctional, pro-atherogenic particle (44). Similarly, calprotectin (S100A8/A9) can signal through Toll-like receptor 4 (TLR4) on adipocytes and skeletal muscle cells to exacerbate systemic metabolic stress (45). Therefore, these protein profiles capture the concurrent progression of articular destruction and systemic metabolic decay (46).

Emerging evidence points to the role of long non-coding RNAs (lncRNAs) in PsA pathogenesis. Dolcino et al. identified lncRNAs that target genes involved in immune response, glycolipid metabolism, bone remodeling, and TNF-α signaling in PsA patients (47). These lncRNAs may regulate both microRNAs and protein-coding genes, and their expression profiles could serve as biomarkers for disease progression (47). The integration of lncRNA profiling with other omics data may enhance predictive modeling. While the aforementioned immunological markers provide valuable mechanistic insights, a critical evaluation of the current literature reveals significant translational limitations. A major confounding factor is that most studies—such as those identifying elevated ASC, IL-18, and calprotectin—utilize strictly cross-sectional designs, comparing established PsA with PsO. Consequently, it remains difficult to determine whether these markers actively drive the transition to PsA (acting as the “tipping point”) or if they are simply epiphenomena of an already established systemic disease. Furthermore, markers like calprotectin appear to reflect generalized systemic inflammation rather than joint-specific progression (40). To date, true predictive value has only been convincingly demonstrated through longitudinal tracking, exemplified by the declining trajectory of CXCL10 prior to PsA onset (35). Therefore, future biomarker research must prioritize prospective, longitudinal study designs to validate the predictive supremacy of these immunological targets.

## 4 Metabolic biomarkers and adipokine signatures in the transition from PsO to PsA

Metabolic dysfunction is intimately linked to the pathogenesis and progression of psoriatic disease. The high prevalence of metabolic syndrome components—obesity, insulin resistance, dyslipidemia, and hypertension—in PsA patients compared to the general population and to PsO-only patients suggests that metabolic alterations may contribute to the transition from cutaneous to articular disease (6, 8, 19, 48–50). Meta-analyses have confirmed that PsA patients have a higher prevalence of metabolic syndrome compared to RA and ankylosing spondylitis patients, and a trend toward higher prevalence compared to PsO-only patients (50–52).

Obesity is the most well-established metabolic risk factor for PsA development. Multiple cohort studies have demonstrated that increasing body mass index (BMI) is associated with a dose-dependent increase in PsA risk among PsO patients (30, 31). Love et al. found that compared to PsO patients with BMI < 25 kg/m², the relative risk of developing PsA was 1.09 for BMI 25-29.9, 1.22 for BMI 30-34.9, and 1.48 for BMI ≥ 35 (31). Obesity at age 18 years predicts PsA risk over and above current BMI, suggesting that early-life adiposity programs long-term inflammatory susceptibility (30).

The mechanistic link between obesity and PsA involves multiple interconnected pathways. Adipose tissue dysfunction in obesity leads to altered adipokine secretion, with increased levels of pro-inflammatory adipokines (leptin, resistin, visfatin) and decreased levels of anti-inflammatory adipokines (adiponectin, omentin) (19, 21, 29, 53). Leptin promotes Th17 differentiation and IL-17 production, inhibits Treg function, and stimulates keratinocyte proliferation (19, 21, 54). Belli et al. demonstrated that circulating leptin levels are higher in female psoriatic patients and correlate positively with PASI score, adiposity indices, and the presence of PsA (54). Higher baseline leptin levels were also associated with failure to achieve PASI90 response to anti-IL-23 therapy, suggesting that leptin may serve as a predictive biomarker for treatment outcomes (54).

Adiponectin, in contrast, exerts anti-inflammatory effects by inhibiting NF-κB activation and promoting Treg differentiation (21, 29). Serum adiponectin levels are decreased in PsO and PsA patients, and this reduction correlates with disease activity and metabolic abnormalities (21, 29, 55). The ratio of leptin to adiponectin, which reflects the balance between pro- and anti-inflammatory adipokine signals, is elevated in PsA and may be a more informative biomarker than either adipokine alone (21, 56).

Resistin and visfatin are pro-inflammatory adipokines that have been investigated in psoriatic disease. Resistin levels are elevated in PsA and correlate with inflammatory markers such as IL-6 (53, 57). Resistin may contribute to insulin resistance and endothelial dysfunction, linking adipose tissue inflammation to cardiovascular risk (53). Visfatin levels are also elevated in PsO and PsA and may promote inflammation through NAD+ biosynthesis and cytokine induction (57, 58). Omentin-1, an anti-inflammatory adipokine, is decreased in PsO and PsA and may have protective effects against metabolic dysfunction (53, 58).

Insulin resistance is a central feature of the metabolic dysfunction associated with PsA. Haroon et al. found that insulin resistance, defined by homeostatic model assessment for insulin resistance (HOMA-IR) > 2.5, was present in 16% of PsA patients and was independently associated with more severe PsA, later PsO age of onset, and higher BMI (59). Bellan et al. demonstrated that inflammatory markers predict insulin sensitivity in active RA but not in PsA, suggesting disease-specific mechanisms linking inflammation and metabolism (60). The relationship between insulin resistance and PsA disease activity perfectly exemplifies the bidirectional immunometabolic loop: in the immune-to-metabolism direction, systemic cytokine spillover (e.g., TNF-α, IL-17) directly interferes with intracellular insulin signaling cascades in peripheral tissues; simultaneously, in the metabolism-to-immune direction, the resulting hyperglycemia and compensatory hyperinsulinemia persistently activate the mTOR pathway in T cells, forcefully amplifying Th17-driven inflammation (61–63).

Dyslipidemia is another hallmark of the metabolic syndrome in PsA. Patients with PsA exhibit characteristic lipid abnormalities, including elevated triglycerides, decreased HDL cholesterol, and altered lipoprotein particle composition (64–66). These changes are associated with increased cardiovascular risk and may also directly contribute to inflammatory pathways (64, 65). The lipid profile in PsA differs from that in RA, with PsA patients showing higher triglyceride levels and lower HDL cholesterol (67). Tecer et al. found that atherogenic indices such as the triglyceride/HDL ratio and the atherogenic index of plasma correlate with carotid intima-media thickness in PsA, suggesting that these simple lipid measures may capture cardiovascular risk (66).

Serum uric acid levels are elevated in PsO and PsA and may contribute to disease pathogenesis through multiple mechanisms. Gisondi et al. demonstrated that hyperuricemia is more prevalent in PsO patients than in matched controls, and this association was independent of metabolic syndrome features (68). Urate is the end-product of purine metabolism in humans. While it has been suggested that urate can act as a free radical scavenger with natural antioxidant properties, the majority of evidence indicates that it functions predominantly as a pro-inflammatory molecule in the context of metabolic dysregulation. In the absence of monosodium urate (MSU) crystal formation—which characterizes gout—soluble urate acts as a potent metabolic danger signal. Soluble urate has been shown to induce long-term epigenetic reprogramming in myeloid cells (trained immunity) and increase oxidative stress by stimulating NADPH oxidase-dependent reactive oxygen species (69). Therefore, in the PsO to PsA transition, it is the systemic burden of this pro-inflammatory soluble urate, rather than crystal deposition, that exacerbates immune dysregulation and drives disease progression (70). In PsA, elevated uric acid levels correlate with disease activity and may predict radiographic progression (70, 71).

Metabolomic profiling has revealed distinct metabolic signatures associated with PsA. Armstrong et al. identified altered levels of alpha-ketoglutaric acid, asparagine, glutamine, glucuronic acid, and lignoceric acid in PsO and PsA patients compared to controls (72). Angioni et al. found that PsA patients exhibited increased glucose and glycyl proline and decreased alanine, glutamine, methionine, serine, and branched-chain amino acids compared to healthy controls (73). These metabolic alterations suggest disturbances in energy metabolism, oxidative stress, and amino acid metabolism that may be relevant to disease pathogenesis (18, 73).

Lipidomic profiling has identified specific lipid species that differentiate PsA from PsO. Koussiouris et al. found that 18 fatty acid and lipid features were significantly different between PsO and PsA patients using solid-phase microextraction coupled with liquid chromatography-mass spectrometry (74). Elevated levels of long-chain fatty acids, including 3-hydroxytetradecanedioic acid, were observed in severe PsA, while eicosanoids with pro- or anti-inflammatory properties were detected exclusively in moderate to severe PsA (75). These findings suggest that lipid dysregulation is not merely a consequence of systemic inflammation but may actively contribute to disease progression (74, 75).

Bile acid metabolism is altered in PsA and may reflect gut microbial dysbiosis. Paine et al. found that bile acid and butyrate levels were depressed in PsO patients who progressed to PsA compared to those who did not, while inflammatory lipid mediators increased after PsA diagnosis (76). The combination of leukotriene B4 and glycoursodeoxycholic acid sulfate showed sensitivity and specificity for predicting PsA progression (76). These findings highlight the potential role of the gut-joint axis in PsA pathogenesis and suggest that metabolic markers of gut microbial function may serve as predictive biomarkers (76, 77). Rather than isolated occurrences, these metabolic disturbances—particularly the dynamic shifts in the leptin-to-adiponectin ratio and altered lipidomic profiles—serve as direct, systemic readouts of the immunometabolic tipping point. However, critically appraising this field reveals that the current metabolomic and lipidomic literature in psoriatic disease is heavily reliant on small-scale exploratory cohorts. A critical bottleneck is distinguishing causality: whether the observed metabolite alterations (such as depleted bile acids or specific long-chain fatty acids) are causal drivers that precipitate joint inflammation, or merely compensatory metabolic responses to chronic systemic stress. Overcoming this requires transitioning from descriptive metabolic profiling to mechanistic validation and prospective cohort testing (**Table 1**).

**Table 1:** Summary of candidate immunometabolic biomarkers in the transition from PsO to PsA.

Category	Specific biomarker	Observed trend in PsA/transition	Mechanistic/clinical relevance	References
Immunological	CXCL10	Declining (in serum prior to PsA onset)	Longitudinal predictive value; suggests consumption or sequestration at sites of developing joint inflammation	(35)
Immunological	CD8+CCR10+ T cells	Elevated (in circulation)	Reflects the systemic recirculation of cutaneous tissue-resident memory (TRM) cells to joints	(25)
Immunological	ASC & IL-18	Elevated (in serum of PsO vs. healthy controls)	Cross-sectional markers of inflammasome activation; drives systemic metabolism disruption	(39)
Immunological	Calprotectin (S100A8/A9)	Elevated (in plasma of PsA vs. controls)	Reflects generalized systemic inflammation rather than localized ultrasound-detected synovitis	(40)
Immunological	Type I IFN score	Lower (in PBMC of PsA vs. healthy controls)	Distinguishes PsA from other inflammatory arthritides (e.g., RA, SLE) where IFN score is typically high	(38)
Immunological	IL-6, SAA, YKL-40, ICAM-1	Elevated (in serum)	Correlate with composite disease activity and exert systemic metabolic effects (e.g., insulin resistance).	(41)
Metabolic	Leptin	Elevated	Pro-inflammatory adipokine; promotes Th17 polarization and inhibits Treg suppressive function	(54)
Metabolic	Adiponectin	Decreased	Loss of anti-inflammatory regulation and systemic metabolic homeostasis	(21, 29, 55)
Metabolic	Leptin-to-Adiponectin Ratio	Elevated	A superior integrated metric reflecting the balance of pro- and anti-inflammatory adipokine signals	(21, 56)
Metabolic	Uric Acid	Elevated	Metabolic danger signal; activates the NLRP3 inflammasome and stimulates pro-inflammatory cytokines	(68–71)
Metabolic	Long-chain fatty acids (e.g., 3-hydroxytetradecanedioic acid)	Elevated/Altered	Cross-sectional lipidomic signatures that actively contribute to, or reflect, systemic inflammatory disease progression	(74, 75)
Metabolic	Secondary bile acids & Butyrate	Depressed (prior to PsA onset)	Fecal/serum metabolites reflecting gut microbiome dysbiosis along the gut-joint axis	(76, 78)

## 5 Integration of multi-omics approaches for predictive modeling

The complexity of psoriatic disease, with its interplay of genetic, immunological, and metabolic factors, necessitates integrated approaches that capture multiple dimensions of biology. Single-omics analyses have identified candidate biomarkers, but their predictive performance has been limited. Multi-omics integration, combining genomics, transcriptomics, proteomics, metabolomics, and epigenomics, offers the potential to develop robust predictive models that can identify PsO patients at high risk of PsA development (23, 24, 79).

Genetic studies have identified numerous susceptibility loci for PsO and PsA, but few variants differentiate between the two conditions. Genome-wide association studies (GWAS) have implicated genes involved in the IL-23/Th17 pathway, NF-κB signaling, and antigen presentation (13–15). HLA-Cw6 is strongly associated with early-onset PsO, while HLA-B27 is uniquely linked to PsA (13, 80). The IL23R locus has been associated with both PsO and PsA, but different variants may predispose to each condition (14, 15). Polygenic risk scores combining multiple genetic variants may improve risk prediction but currently lack sufficient discriminatory power for clinical use (15, 23).

Epigenetic modifications, including DNA methylation and histone modifications, provide a mechanism through which environmental factors influence gene expression in psoriatic disease. Natoli et al. demonstrated that DNA methylation patterns in CD4+ T cells discriminate between PsO and PsA patients (81). Differentially methylated positions affected genes involved in wound healing, cell adhesion, and cAMP-dependent protein kinase activity (81). Treatment with cytokine inhibitors partially corrected DNA methylation patterns, and methylation scores correlated with skin disease activity (81). These findings suggest that epigenetic markers may capture disease activity and progression risk.

Transcriptomic profiling has identified gene expression signatures that differentiate PsA from PsO. Single-cell transcriptomic analysis has revealed the metabolic features of T cell subsets in psoriatic disease, including CD4+ central memory T cells, CD8+ effector memory T cells, Tregs, mucosa-associated invariant T (MAIT) cells, and γδ T cells (17). Peng et al. found that upregulation of ascorbate and aldarate metabolism and fatty acid degradation may enhance Treg suppressive function, while enhanced polyunsaturated fatty acid metabolism may inhibit pro-inflammatory functions of CD4+ and CD8+ T cells (17). These findings suggest that metabolic pathways are differentially regulated in distinct T cell subsets and may be targeted for therapeutic intervention (17).

Proteomic profiling has identified panels of proteins that may distinguish PsA from other arthritides and predict disease progression. Chandran et al. found that a panel of four biomarkers—COMP, resistin, MCP-1, and NGF—distinguished PsA from osteoarthritis with an AUC of 0.99 (82). Souto-Carneiro et al. developed a multivariate diagnostic model incorporating age, sex, alanine, succinate, creatine phosphate, and lipid ratios that discriminated seronegative RA from PsA with an AUC of 84.5% (83). These protein panels may be useful for early diagnosis of PsA in patients presenting with arthritis.

Metabolomic profiling has emerged as a tool for identifying biomarkers of PsA. Nuclear magnetic resonance spectroscopy and mass spectrometry have been used to characterize the serum metabolome in psoriatic disease (18, 22, 84). Kishikawa et al. performed large-scale plasma metabolome analysis and identified ethanolamine phosphate as significantly increased in PsO, while tyramine and mucic acid differed between PsA and cutaneous PsO (85). Pathway analysis revealed enrichment of vitamin digestion and absorption pathways in cutaneous PsO (85).

Fecal metabolomics has revealed alterations in amino acid, bile acid, and lipid metabolism in PsA. Wang et al. identified 154 fecal metabolites significantly altered in PsA compared to healthy controls, and 45 metabolites different from RA (78). A support vector machine model based on five fecal metabolites—α/β-turmerone, glycerol 1-hexadecanoate, dihydrosphingosine, pantothenic acid, and glutamine—distinguished PsA from RA and healthy controls (78). These findings suggest that gut microbial metabolites may serve as non-invasive biomarkers for PsA.

Lipidomics has identified specific lipid species associated with PsA activity and progression. Choksi et al. found that serum metabolites associated with skin disease activity in PsA included eicosanoids with anti- or pro-inflammatory properties, bile acids, oxidized phospholipids, and long-chain fatty acids (86). Myśliwiec et al. demonstrated altered serum fatty acid profiles in PsA, with higher percentages of saturated and monounsaturated fatty acids and lower polyunsaturated fatty acids compared to controls (87). The ratio of saturated to unsaturated fatty acids was higher in PsA than in PsO, suggesting that lipid metabolism is differentially altered in articular disease (87).

Sphingolipid metabolism is disrupted in psoriatic disease. Myśliwiec et al. found that serum ceramide levels were decreased while sphingosine-1-phosphate levels were increased in PsO patients compared to controls (88). Patients with PsA had higher ceramide levels than those with skin-only PsO, suggesting that ceramide metabolism may be differentially regulated in articular disease (88). Sphingolipids are involved in cell growth, apoptosis, and immune regulation, and their dysregulation may contribute to both skin and joint pathology.

While the aforementioned studies highlight the individual power of single-omics platforms, the true paradigm shift in forecasting the immunometabolic tipping point relies on the simultaneous integration of these multi-omic layers (89). Recently, pioneering studies have begun to adopt true integrative frameworks in psoriatic disease (90). For instance, the integration of metagenomics (microbiome) and untargeted metabolomics has been instrumental in elucidating the gut-joint axis (91). By linking altered fecal microbial composition directly to the systemic depletion of anti-inflammatory metabolites—such as secondary bile acids and short-chain fatty acids—researchers have identified cross-omic networks that drive PsA progression (92). Furthermore, integrative approaches combining genomics (GWAS) with transcriptomics (eQTLs) have successfully bridged the gap between PsA-specific genetic risk loci and their functional downstream effects on gene expression in distinct T cell subsets (93). Similarly, combining single-cell transcriptomics (scRNA-seq) with high-throughput spatial proteomics of synovial and cutaneous tissues is providing a multidimensional map of how metabolic reprogramming in localized TRM cells correlates with systemic cytokine gradients. These integrated approaches do not merely summate data; they unveil causal biological networks and complex molecular interactions that isolated single-omics analyses inherently miss (94).

Machine learning and artificial intelligence (AI) approaches are increasingly being applied to integrate multi-omics data for predictive modeling in psoriatic disease (**Figure 2**). Random forest models, support vector machines, and neural networks can identify complex patterns across omics layers that are not apparent from univariate analyses (23, 24). Kulyk and De Vlam discussed how integrating metabolic endotypes, immunological signatures, genetic markers, and clinical data through machine learning may facilitate earlier diagnosis and personalized therapy in PsA (23).

**Figure 2:**
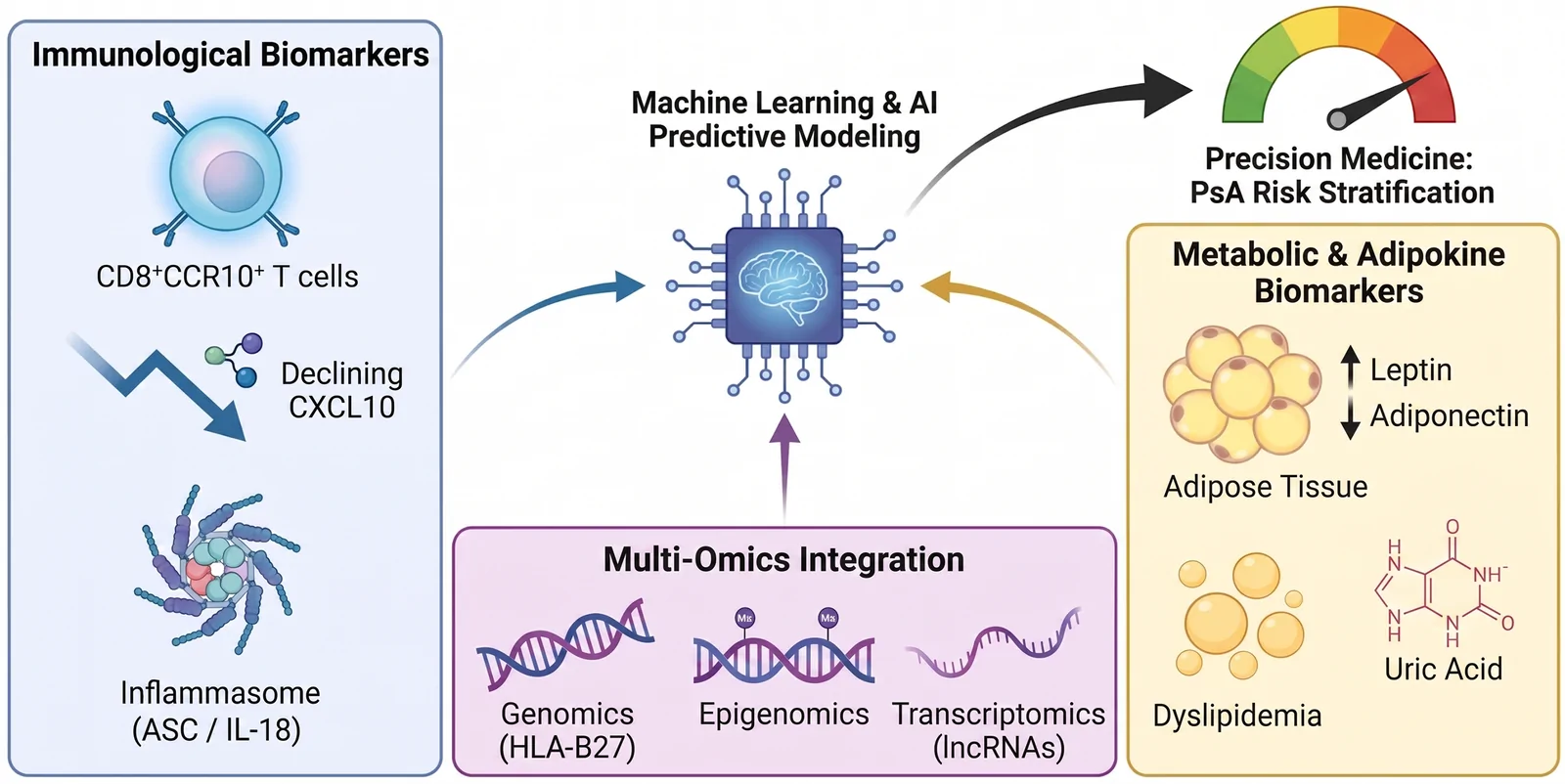
Conceptual framework for predicting psoriatic arthritis through the integration of multi-omics and immunometabolic biomarkers. The transition from psoriasis (PsO) to psoriatic arthritis (PsA) is driven by a complex interplay of immunological and metabolic dysregulation. Left panel: Immunological candidate biomarkers predicting PsA onset include the expansion of circulating skin-homing CD8+CCR10+ T cells, declining serum CXCL10 levels (indicating potential sequestration at inflammatory sites), and markers of inflammasome activation (ASC and IL-18). Right panel: Metabolic and adipokine signatures are characterized by adipose tissue dysfunction (elevated leptin and decreased adiponectin levels), systemic dyslipidemia, and elevated uric acid. Bottom panel: Multi-omics integration captures the multidimensional pathogenesis of psoriatic disease, encompassing genomics (e.g., HLA-B27 susceptibility), epigenomics (e.g., DNA methylation alterations), and transcriptomics (e.g., lncRNAs). Center and Top Right: Synthesizing these diverse, high-dimensional datasets through machine learning and artificial intelligence (AI) predictive modeling enables robust risk stratification. This data-driven approach facilitates preemptive interventions and advances precision medicine in the management of patients at high risk of PsA development.

The HIPPOCRATES project, a European IMI2 initiative, exemplifies the multi-omics approach to PsA biomarker discovery. This project integrates genomic, epigenomic, transcriptomic, proteomic, metabolomic, and lipidomic data with clinical and imaging data to define biomarker profiles specific for PsA (24). The goal is to develop molecular profiles that can diagnose PsA, predict its occurrence in PsO cohorts, differentiate PsA from other inflammatory arthritides, and improve therapeutic responses (24). Moving beyond its initial conceptualization, the consortium has recently achieved critical milestones. Most notably, it has launched the HIPPOCRATES Prospective Observational Study (HPOS), a large-scale, digital-first longitudinal cohort actively tracking thousands of PsO patients to capture the real-time clinical and molecular transition to PsA. Preliminary phases of the project have successfully focused on the rigorous harmonization of extensive retrospective multi-omics datasets across multiple European centers. By establishing standardized bioinformatics pipelines to handle batch effects and high-dimensional data integration, these foundational efforts are currently yielding early composite molecular signatures that are now entering prospective validation phases.

Despite the transformative promise of multi-omics and AI-driven modeling, a critical appraisal highlights several methodological and translational hurdles. The integration of high-dimensional omics data using machine learning is inherently susceptible to the “curse of dimensionality” and severe model overfitting, particularly given the relatively small sample sizes typically available in current PsA cohorts. Many proposed multi-omics algorithms demonstrate excellent performance in training datasets but fail to generalize. Furthermore, the integration of data across omics layers requires sophisticated bioinformatics to handle multiple testing and batch effects (79). Therefore, while AI models excel at mapping the immunometabolic tipping point in exploratory settings, transitioning these predictive tools into clinical practice fundamentally demands rigorous validation in independent, large-scale, prospective, and multi-center cohorts (15, 23).

## 6 Conclusion and future perspectives

The transition from PsO to PsA represents a critical window for intervention, yet the ability to predict which patients will develop articular disease remains limited. Immunometabolic biomarkers offer a promising avenue for improving risk stratification, as they capture the intersection of immune dysregulation and metabolic dysfunction that drives disease progression. The IL-23/Th17 axis, TRM cells, and Treg dysfunction constitute the immunological core of psoriatic disease, while obesity, insulin resistance, dyslipidemia, and adipokine imbalance provide the metabolic context that amplifies inflammation and may facilitate the extension from skin to joint pathology.

Several candidate biomarkers have shown promise for predicting PsA onset. Declining CXCL10 levels, expansion of CD8+CCR10+ T cells, altered adipokine profiles (particularly leptin and adiponectin), and specific metabolomic and lipidomic signatures warrant further investigation in prospective studies. The combination of biomarkers from multiple domains, integrated through machine learning approaches, may achieve the predictive performance needed for clinical implementation.

The clinical implications of identifying high-risk PsO patients are substantial. Early intervention with disease-modifying therapies could potentially prevent or delay the onset of PsA, preserving joint function and preventing irreversible damage (3, 5). Lifestyle interventions targeting obesity and metabolic dysfunction may reduce PsA risk and improve treatment outcomes (6, 32, 33). The Interception in Very Early Psoriatic Arthritis (IVEPSA) and Preventing Arthritis in a Multicentre Psoriasis At-Risk cohort (PAMPA) studies, which aim to improve knowledge regarding PsA interception in high-risk PsO patients, represent important steps toward this goal (3).

Several knowledge gaps remain. The precise mechanisms through which metabolic dysfunction promotes the transition from cutaneous to articular inflammation are incompletely understood. The role of the gut microbiome and its metabolic products in PsA pathogenesis requires further investigation (16, 76, 95). Sex differences in PsA presentation and progression suggest that sex-specific biomarkers and risk prediction models may be needed (57, 96–98). The effects of different therapeutic agents on immunometabolic profiles and their relationship to long-term outcomes warrant study.

Future research should prioritize longitudinal cohort studies with comprehensive phenotyping at multiple time points during the transition from PsO to PsA. Furthermore, because metabolic signatures and lipidomic profiles are inherently susceptible to chronological shifts, strictly accounting for these confounding variables is paramount. Developing age-adjusted biomarkers and age-stratified predictive models will be critical to ensure diagnostic accuracy. Integration of multi-omics data with clinical (including critical demographic confounders such as age and sex), imaging, and environmental factors will be essential for developing robust predictive models. Validation of candidate biomarkers in diverse populations and standardization of analytical methods will facilitate clinical translation. The development of point-of-care assays for key biomarkers could enable widespread screening of PsO patients.

The goal is to achieve precision medicine in psoriatic disease, where individual patients are stratified according to their risk of PsA development and treatment is tailored accordingly. Immunometabolic biomarkers, capturing the dynamic interplay between inflammation and metabolism, are uniquely positioned to contribute to this vision. As understanding of the mechanisms driving the systemic shift from PsO to PsA deepens, the prospect of preventing articular disease in at-risk patients moves closer to clinical reality.
